# Comparing image normalization techniques in an end-to-end model for automated modic changes classification from MRI images

**DOI:** 10.1016/j.bas.2023.102738

**Published:** 2023-12-23

**Authors:** Andrea Cina, Daniel Haschtmann, Dimitrios Damopoulos, Nicolas Gerber, Markus Loibl, Tamas Fekete, Frank Kleinstück, Fabio Galbusera

**Affiliations:** a ETH Zürich, Department of Health Sciences and Technologies, Zürich, Switzerland; b Schulthess Klinik, Department of Teaching, Research and Development, Zürich, Switzerland; c Schulthess Klinik, Department of Spine Surgery and Neurosurgery, Zürich, Switzerland; d University of Bern, Bern, Switzerland; e Personalised Medicine Research, School of Biomedical and Precision Engineering, University of Bern, Switzerland

**Keywords:** Modic changes, MRI images, Deep learning, Automatic classification, Detection

## Abstract

**Introduction:**

Modic Changes (MCs) are MRI alterations in spine vertebrae's signal intensity. This study introduces an end-to-end model to automatically detect and classify MCs in lumbar MRIs. The model's two-step process involves locating intervertebral regions and then categorizing MC types (MC0, MC1, MC2) using paired T1-and T2-weighted images. This approach offers a promising solution for efficient and standardized MC assessment.

**Research question:**

The aim is to investigate how different MRI normalization techniques affect MCs classification and how the model can be used in a clinical setting.

**Material and methods:**

A combination of Faster R–CNN and a 3D Convolutional Neural Network (CNN) is employed. The model first identifies intervertebral regions and then classifies MC types (MC0, MC1, MC2) using paired T1-and T2-weighted lumbar MRIs. Two datasets are used for model development and evaluation.

**Results:**

The detection model achieves high accuracy in identifying intervertebral areas, with Intersection over Union (IoU) values above 0.7, indicating strong localization alignment. Confidence scores above 0.9 demonstrate the model's accurate levels identification. In the classification task, standardization proves the best performances for MC type assessment, achieving mean sensitivities of 0.83 for MC0, 0.85 for MC1, and 0.78 for MC2, along with balanced accuracy of 0.80 and F1 score of 0.88.

**Discussion and conclusion:**

The study's end-to-end model shows promise in automating MC assessment, contributing to standardized diagnostics and treatment planning. Limitations include dataset size, class imbalance, and lack of external validation. Future research should focus on external validation, refining model generalization, and improving clinical applicability.

## Highlights

• Automating the detection and classification of Modic Changes (MCs) in MRI.
• Combination of Faster R–CNN and 3D Convolutional Neural Network (CNN) for MC classification.
• Impact of different image normalization techniques on the performance of the classification model.
• Standardized MC assessment, aiding in diagnosis, treatment planning, and research.

## 1 Introduction

The term Modic Changes (MCs) refers to alterations in the signal intensity observed within the vertebral body and endplates in T1-and T2-weighted MRI scans of the spine. The concept of MCs was initially introduced in a few studies from 1987 to 1988 (Assheuer et al., 1987; Modic et al., 1988a). The nomenclature “Modic Changes” was later coined, and a few subsequent works elaborated on the classification of MCs into three distinct patterns and explored their potential link to disc degeneration (de Roos et al., 1987; Modic et al., 1988b). The classification encompasses three distinct MC types, each characterized as follows: MC‐1 is related to a reactive or inflammatory alteration within the bone marrow; MC‐2 is identified as marrow replacement by fat tissue; and MC‐3 is defined by the presence of calcification within the endplate and subchondral vertebral marrow (Jensen et al., 2008; Bierry et al., 2016; Zehra et al., 2018). However, there is still a debate on the precise definition of MCs. In fact, some studies discussed whether Modic changes can consistently evolve from one type to another and can also exist at the same time in the same vertebral body (Albert et al., 2008). A previous study discussed the lack of agreement and clarity in defining MCs highlighting that the term “MC” has been used with varying interpretations in the literature, leading to inconsistencies in diagnostic criteria, variations in study populations, and conflicting results (Udby et al., 2022).

Additionally, the authors proposed a structured grading system to categorize different types of MCs and quantify their impact on vertebral bodies. The authors stressed that MC holds clinical relevance, underscoring the need for standardizing the language, definition, and grading of MC to enhance consistency and accuracy in research and clinical studies. A few studies examined the association between MCs and Low Back Pain (LBP). In fact, MC‐1 and MC‐2 have been suggested as potential contributors to significant pain (Jensen et al., 1994; Kääpä et al., 2012; Herlin et al., 2018). Notably, a study conducted by Kjaer et al. (2006) highlighted the connection between MCs and disc degeneration on MRI, showcasing an increased likelihood of experiencing (LBP) in a cohort of 40‐year‐old individuals from Denmark. This correlation was found also in another paper where the authors proposed a new endplate lesions classification scheme using a population affected by LBP (Brayda-Bruno et al., 2018).

Given that Modic Changes are evaluated through imaging, it is logical to consider that the progress in artificial intelligence (AI) could be exploited for the automated detection and classification of MCs in MRI. The aim of this study was to create a model that can automatically find intervertebral areas and classify each level based on the presence of Modic Changes (MCs) into three categories: no MC (MC0), type 1 (MC1), and type 2 (MC2), based on paired T1-and T2-weighted lumbar sagittal MRIs. To achieve this, a combination of a detection model called Faster R–CNN and a 3D classification model was used. The first model helped to locate specific regions of interest (ROIs) within the complex T2-weighted lumbar MRIs. Then, the second model, a 3D Convolutional Neural Network (CNN) - was applied to classify each ROIs. Our end-to-end approach aims to effectively tackle the challenge of automated intervertebral area identification and the subsequent classification of different MC types.

## 2 Materials and methods

### 2.1 Datasets

In this study, two datasets of MRI images collected from 2 different hospitals have been used. All subjects gave informed consent for scientific and educational use of the images. The first set contained T2-weighted MRI images from 761 patients. Each image was annotated with the x-y coordinates of the bounding box that localized the intervertebral discs from T12/L1 to L5/S1. For the model development and evaluation, the dataset was split into 80% for training and 20% for testing. The second dataset came from an earlier study (Damopoulos et al., 2019) and contained both T1-and T2-weighted MRI images. This dataset included images from 912 patients labeled according to the MCs classification scheme. The distribution of instances among the three classes was extremely imbalanced, with the percentages being 80% for MC0, 5% for MC1, and 15% for MC2 respectively. Therefore, due to the pronounced class imbalance, a specific approach was employed, namely stratified cross-validation to maintain consistent proportions of the three classes across distinct folds. Specifically, through a 5-fold cross-validation strategy that was applied 10 times (repeated stratified K-fold).

### 2.2 Deep learning models

Two distinct deep learning models were developed. The first model was trained using the first dataset with the aim to localize and label each lumbar vertebra from the central slice of a T2-weighted MRI image. The Faster R–CNN model presented in (Ren et al., 2015) was used by applying a technique called Transfer Learning where pretrained models trained on huge datasets are adapted to domain-specific tasks. For our study, a ResNet50 (He et al., 2015) was used as the backbone. The model was trained to determine the bounding box coordinates for each level, associate the bounding box with its respective level label, and assign a confidence score to the labeling. Since a level can be detected multiple times, namely multiple bounding boxes with different confidence scores might be associated with the same vertebral levels, the highest confidence score was used to extract each level only once. This ensures a maximum of six regions of interest (ROIs) from T12/L1 to L5/S1.

**Fig. 1:**
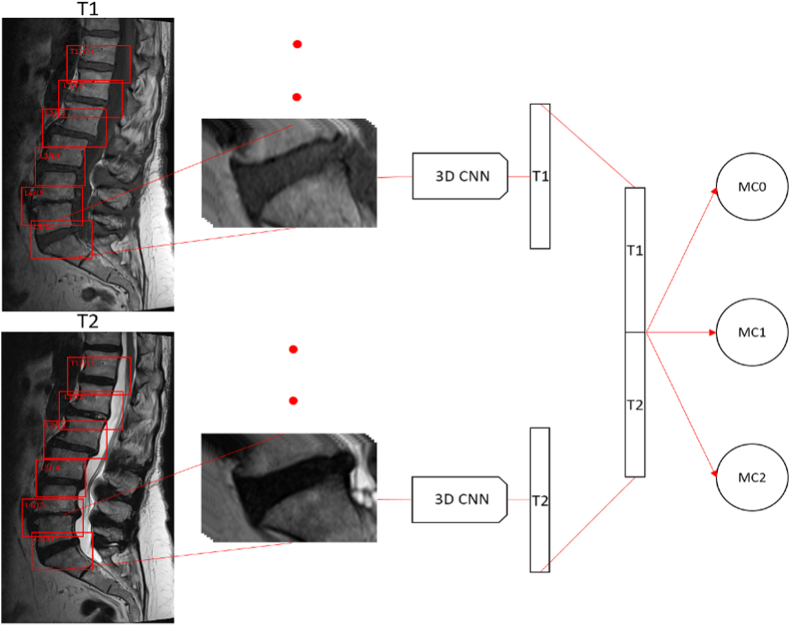
**End-to-end model**. The vertebral levels are extracted from T1-and T2-weighted MRI images to obtain five slices for each vertebral level that are fed to the 3D classification model. The model extracts two feature vectors, one for T1 and one for T2, concatenates them, and classifies the slices into the three MC types.

The second model was trained using the second dataset. First, the extraction of ROIs was achieved through our Faster R–CNN detection model. This involved processing T1-and T2-weighted images through our Faster R–CNN, resulting in the identification of six ROIs per image. Each ROI consisted of five slices, symmetrically extracted around the midsagittal slice. The slices were processed by two identical 3D CNN models to extract the respective feature vectors which were then concatenated to predict the MC (Fig. 1). This was done to be consistent with the human evaluation where T1-and T2-weighted images are evaluated simultaneously to determine MC classification. Going deeper, a 3D version of the ResNet network was used exploiting the utilities developed by the project Medical Open Network for Artificial Intelligence (MONAI) (Jorge Cardoso et al., 2022). MONAI is an open-source domain-specific framework designed to facilitate the development and application of deep learning techniques in medical image analysis. Thus, the network's last layer was modified to have three output neurons to classify into the three MCs. Transfer Learning was used also for this task but relying on the model parameters computed in a study that collected many different 3D medical image datasets to have a general model that can be finetuned for specific tasks (Chen et al., 2019). The Med3D model offers transfer learning for 3D medical image analysis, enabling efficient adaptation and fine-tuning of pre-trained models to enhance performance and address data limitations in medical imaging tasks.

The Faster R–CNN model was trained using the mean of the losses resulting from the different tasks of the model; namely, the L1 loss between the predicted box coordinates and the ground truth box coordinates, a cross-entropy loss to find whether each region proposal contains an object or not, and the softmax cross-entropy loss to optimize the class label of the object within each region proposal. The second model was trained using a cross-entropy loss after applying a softmax function to the network output. All the models were optimized using the Adam optimizer starting from a learning rate of 0.001 and early stopping was used to stop the training if the validation loss did not improve for 5 epochs. Image augmentation techniques including rotations between −10 and 10° and shifts (−10 to 10 pixels) were used to increase the robustness of the models. The code was implemented in Python using PyTorch^1^ for models’ development, open-CV^2^ for image processing, and Albumentations^3^ for image augmentation.

### 2.3 Evaluation

For the assessment of the detection model performance, the Intersection over Union (IoU) metric was used to quantify the overlap between the predicted bounding boxes and the ground truth annotations, allowing for precise quantification of localization accuracy. Furthermore, a confidence score was assigned to each object prediction contained within the bounding box, contributing to the model's capabilities in identifying vertebral levels. All these metrics were evaluated on the test set.

For the second dataset, repeated (10 times) five-fold cross-validation (CV) was used to have multiple evaluations on different subsets of the main dataset. The four normalization techniques were no normalization, standardization (zero mean and unit variance), CLAHE (Contrast Limited Adaptive Histogram Equalization), and Gamma Correction. In a few words, CLAHE is used to enhance image contrast by locally adjusting pixel values using histograms while limiting amplification and Gamma Correction to adjust image pixel values using a gamma factor to correct brightness and contrast. The latter has been widely used to process MRI images (Sahnoun et al., 2018; Somasundaram and Kalavathi, 2011). The performance metrics were the sensitivities for each class, namely the capability of the model to correctly classify the MC, the balanced accuracy that provides a model's overall performance by considering equal weight to each class, and the F1-score which is a single metric that combines precision and recall.

## 3 Results

Regarding the first dataset, 609 samples were used to train the detection model, reserving 152 for testing purposes. In the context of the second dataset, applying stratified cross-validation yielded a training set of 730 samples distributed over four folds, while the remaining fold encompassed 182 samples designated for model validation. Importantly, this configuration maintained the 80/5/15 distribution ratio among the three MC types. Moving to the performances, the Intersection over Union (IoU) values on the test set showed values above 0.7 indicating high overlap between true and predicted bounding boxes. The confidence scores were all above 0.9 demonstrating high confidence in levels classification (Table 1).

**Table 1:** **Performance of the detection model**. The IoU above 0.7 indicate a high overlap between the true and the predicted bounding boxes. The confidence scores are all above 0.9 showing high confidence in levels identification.

Metric	Intervertebral level					
	T12/L1	L1/L2	L2/L3	L3/L4	L4/L5	L5/S1
IoU	0.7	0.8	0.78	0.81	0.82	0.81
Confidence score	0.93	0.98	0.98	0.93	0.97	0.98

IoU measurements above 0.7 indicate strong alignment between predicted bounding boxes and ground truth, while high confidence scores demonstrate the model's accurate extraction of Regions of Interest (ROIs) from medical images, ensuring reliable extraction in the second dataset.

The in-depth evaluation of various normalization techniques identified standardization in general as the optimal choice for all evaluation metrics. In general, not applying any normalization seems to lead to poor results.

**Fig. 2:**
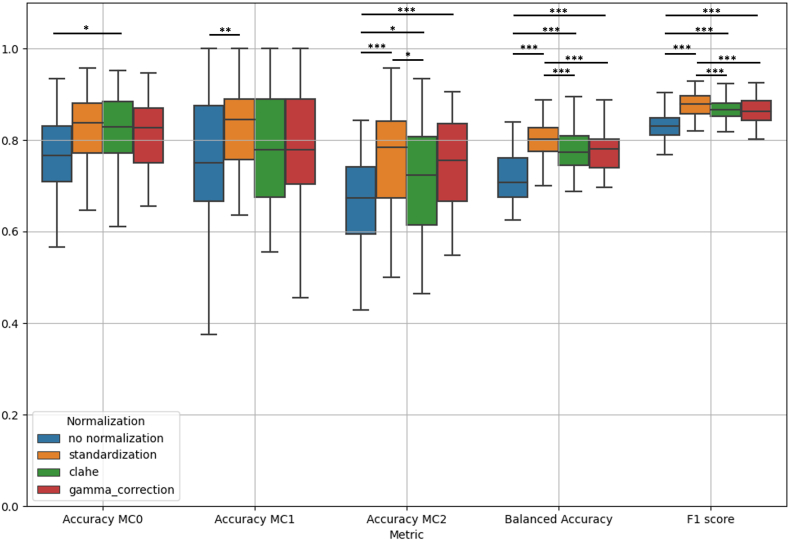
**Performance metrics.** Boxplot of MC types classification accuracies, balanced accuracy, and F1 score of the 10 repeated 5 folds stratified by normalization (colors). *p < 0.05, ** 0.01 < p < 0.05, ***p ≪ 0.01. (For interpretation of the references to color in this figure legend, the reader is referred to the Web version of this article.)

Looking at standardization performances, the mean sensitivities of 0.83 (SD 0.08), 0.85 (SD 0.12), and 0.78 for MC0, MC1, and MC2 respectively, highlight the capability of the model in correctly classifying the three types of MCs (Fig. 2). Moreover, the balanced accuracy of 0.80 (SD 0.04) further confirms the model's equilibrium in making accurate predictions across imbalanced classes. The F1 score, evaluated across the different classes, is 0.88 (SD 0.02), emphasizing the model's ability in maintaining a balance between true positives and false positives (Table 2).

**Table 2:** **Performance of the classification model**. Means and standard deviations across the repeated 5-fold cross-validation.

	Accuracy MC-0	Accuracy MC-1	Accuracy MC-2	Balanced accuracy	F1
No normalization	0.76 (0.09)	0.73 (0.14)	0.65 (0.11)	0.71 (0.05)	0.83 (0.03)
Standardization	0.83 (0.08)	0.85 (0.12)	0.78 (0.11)	0.80 (0.04)	0.88 (0.02)
CLAHE	0.82 (0.08)	0.79 (0.11)	0.71 (0.12)	0.77 (0.05)	0.86 (0.02)
Gamma correction	0.81 (0.07)	0.78 (0.13)	0.74 (0.13)	0.77 (0.05)	0.86 (0.03)

## 4 Discussion

In this study, we introduced a comprehensive model that performs two main tasks on paired T1-and T2-weighted sagittal MRI images. The model begins by identifying and separating the intervertebral regions, and subsequently, it categorizes these regions into the various MC classes. Our results demonstrated the power of the 3D CNN in achieving good classification performances. For the vertebral bodies extraction, the Faster R–CNN model presented in (Ren et al., 2015) was used since it provided state-of-the-art performances in object detection tasks. In particular, transfer learning (Bozinovski and Fulgosi, 1976) was used to exploit the pretrained model and adapt it to our task, namely localizing and labeling intervertebral disc spaces from the mid-sagittal slice of a T2-weighted MRI image. Transfer learning from ImageNet (Deng et al., 2009) to the medical domain involves using knowledge gained from a large dataset of general images (ImageNet) to improve the performance of deep learning models on medical tasks (Alzubaidi et al., 2020). Faster R–CNN combines a deep convolutional neural network (CNN) with a region proposal network (RPN) (Ren et al., 2015) to efficiently and accurately locate and classify objects within an image. The intervertebral levels detection was very accurate with IoU and confidence scores for all the levels above 0.7 and 0.9 respectively. IoU measurements exceeding the 0.7 threshold mean robust alignment between the predicted bounding boxes and ground truth annotations. Remarkably elevated confidence scores highlight the model's capability in precisely extracting Regions of Interest (ROIs) from the medical images. This means that the ROIs of the second dataset could be extracted with high reliability.

An extensive exploration of diverse image normalization techniques was undertaken in the context of MCs classification to evaluate their impact on classification performance. Given the preliminary task involving the evaluation of different normalization techniques and the aim to leverage the complete dataset for assessment, a decision was made to not use a train-test split. Conducting repeated cross-validation allowed for an in-depth examination of performance variability across diverse subsets, achieved by analyzing the distribution of each performance metric. It is worth noting the importance of image normalization before the model's classification (Fig. 2). In fact, the positive impact of image normalization techniques, in particular image standardization, on the performance of our model was demonstrated. The mean sensitivities, namely the ability to correctly classify the three MCs, were 0.83, 0.85, and 0.78. Automated Modic Changes classification from MRI images might offer clinical value by aiding diagnosis, treatment planning, and patient management, while minimizing interobserver variability. In fact, the trained model will always provide the same output given the same image. The model can also benefit retrospective studies as it would allow a very quick evaluation of large amounts of images reducing humans' workload in MRI assessments. Moreover, the MC types classification can be used to develop predictive models enhancing insights into disease progression and enabling personalized interventions for better patient outcomes.

Previous studies have tried to automatically classify Modic changes from MRI images. Above all, a group of researchers from Oxford developed SpineNet (Jamaludin et al., 2017), a deep learning model to fully analyze lumbar spine MRI images. However, SpineNet only provides a binary classification namely the presence or absence of MCs without giving any information about the MC type. This is due to the use of T2-weighted images only that does not allow to discriminate MC-1 and MC-2. Subsequently, SpineNet was validated on an external validation set giving an accuracy of around 86% (McSweeney et al., 2023). Similar to our work, another study aimed at classifying MCs into three subtypes namely MC-0, MC-1, and MC-2 (Damopoulos et al., 2019). They tried different approaches obtaining accuracies of 93%, 64%, and 81% in correctly classifying MC-0, MC-1, and MC-2. These results were comparable to our findings but our model was significantly better at identifying MC-1 while their model was better for MC-0. However, our model was able to automatically extract the ROIs for the classification while the model by Damopoulos et al. required a manual extraction. A group of researchers tried to develop a method to quantitatively and consistently identify MCs in lumbar spine MRI images using a two stages approach (Gao et al., 2022). First, an autoencoder was trained to segment vertebral bodies from T1-weighted images. Then, another autoencoder is trained using T1-and T2-weighted images, with radiologists' segmented MCs serving as ground truth. Detected regions are categorized into specific Modic types using signal intensity rules. They found accuracies of 67%, 67%, and 44% in identifying MC-1, MC-2, and MC-3. Our accuracies in identifying MC-1 and MC-2 were 0.83 and 0.85 respectively.

A limitation of the work by Gao et al. (2022) is the use of a rule-based approach to perform the final classification. The use of more advanced machine learning techniques might lead to better performances. Moreover, the use of dilated convolution would allow a precise segmentation of the MC specific region (Wolterink et al., 2017). Besides, our sample size was relatively small, which led to the use of cross-validation for assessing model performance. However, it is important to acknowledge that this approach, even if valid and used, lacks the rigor of an initial train-test split, potentially impacting the model's ability to generalize beyond the dataset.

Furthermore, the inherent imbalance within our dataset, with certain classes being more prevalent than others, introduces a potential source of bias. This skew in class distribution may have contributed to better performance for the more represented class, potentially affecting the overall accuracy and robustness of the model's predictions. Finally, a remarkable aspect absent from our study is the lack of any external validation, which restricts our ability to demonstrate the clinical validity of the model beyond the dataset used. External validation, encompassing independent datasets from other institutions, is fundamental to proving the robustness and generalizability of the model. Overall, a notable concern regarding Modic changes (MC) is their association with potentially inaccurate ground truth due to factors such as the weak signal of MC, their limited presence within a small area of a single endplate, or instances of co-occurrence of both MC types within a single vertebra (Viswanathan et al., 2020). Additionally, situations arise where one type is identified in one endplate and the other type in the opposing endplate. It's worth noting that our study outlines a clear roadmap for future research. This roadmap includes addressing the limitations outlined here, such as pursuing external validation and expanding data collection efforts. While our current study provides valuable insights, these limitations underscore the need for ongoing research to refine and enhance the model's performance and clinical utility. Starting from the paper by Gao et al. (2022) a useful idea would be to add to the classification the identification of the region of the vertebral bodies where the MC is located. This can be achieved using techniques to visualize and understand the decisions made by CNNs in image classification tasks such as GradCAM (Selvaraju et al., 2020), and Class Activation Mapping (Zhou et al., 2015). Moreover, the use of STIR MRI images instead of T2-weighted might be investigated as it is becoming more effective in classifying MCs in clinical practice (Beth Vettiyil). In summary, this paper introduces an automated model to detect and classify Modic Changes (MCs) from MRI images, which are linked to spine conditions. The model shows promise in providing standardized MC assessment, aiding diagnosis and treatment planning. Although challenges exist, the study outlines a path for refining the model's clinical application and utility.

## Declaration of competing interest

The authors declare that they have no known competing financial interests or personal relationships that could have appeared to influence the work reported in this paper.

## Footnotes

1 https://pytorch.org/.

2 https://opencv.org/.

3 https://albumentations.ai/.
